# Mitochondrial mechanisms and therapeutics in ischaemia reperfusion injury

**DOI:** 10.1007/s00467-018-3984-5

**Published:** 2018-06-02

**Authors:** Jack L. Martin, Anja V. Gruszczyk, Timothy E. Beach, Michael P. Murphy, Kourosh Saeb-Parsy

**Affiliations:** 1 Department of Surgery and Cambridge NIHR Biomedical Research Centre, Biomedical Campus, University of Cambridge, Cambridge, CB2 2QQ UK; 2 MRC Mitochondrial Biology Unit, Biomedical Campus, University of Cambridge, Cambridge, CB2 0XY UK

**Keywords:** Acute kidney injury, Children, Ischaemia reperfusion injury, Mitochondria, Reactive oxygen species, Succinate

## Abstract

Acute kidney injury (AKI) remains a major problem in critically unwell children and young adults. Ischaemia reperfusion (IR) injury is a major contributor to the development of AKI in a significant proportion of these cases and mitochondria are increasingly recognised as being central to this process through generation of a burst of reactive oxygen species early in reperfusion. Mitochondria have additionally been shown to have key roles in downstream processes including activation of the immune response, immunomodulation, and apoptosis and necrosis. The recognition of the central role of mitochondria in IR injury and an increased understanding of the pathophysiology that undermines these processes has resulted in identification of novel therapeutic targets and potential biomarkers. This review summarises a variety of therapeutic approaches that are currently under exploration and may have potential in ameliorating AKI in children in the future.

## Acute kidney injury in paediatric nephrology

Acute kidney injury (AKI) remains a major problem in critically unwell children and young adults and is recognised as a major risk factor for the development of chronic kidney disease [1, 2]. The reported incidence of AKI varies between studies; a consequence in part of different case mix of institutions and variable definitions of AKI. In a multinational study, 26.9% of patients admitted to paediatric intensive care units were observed to have AKI. Importantly, in these children, AKI was an independent risk factor for morbidity and mortality, highlighting the urgent need for the development of effective therapies to prevent and treat AKI [3, 4]. Ischaemia reperfusion (IR) injury is a major contributor to the development of AKI in a significant proportion of children. Mitochondria are increasingly recognised to have a fundamental role in the pathogenesis of IR injury, and with the development of novel mitochondria-targeted therapies, there is increasing interest in the application of such therapies to the management of AKI [5].

## Ischaemia reperfusion injury and reactive oxygen species

Reperfusion injury is the paradoxical, pathological exacerbation of tissue injury that occurs on re-oxygenation of an organ that has previously been subjected to a period of ischaemia [6]. While early research into this process focused on the heart [7–10], it is now increasingly recognised that the underlying pathophysiological process is common to a wide range of disorders, including AKI, stroke, intestinal ischaemia, multi-organ failure, hypovolaemic shock and organ dysfunction after transplantation [6].

It is well recognised that the dominant injurious effector upon reperfusion is an early burst of reactive oxygen species (ROS). Mitochondria are increasingly recognised as the key source of these ROS, through the generation of a burst of superoxide upon reperfusion, and these findings have been corroborated across a range of tissue types, including renal tissue [11, 12]. Whilst there are a number of alternative sources of superoxide, including the xanthine oxidase pathway and NADPH oxidases that are thought to be important in renal IR injury [13], activation of these pathways seems to occur after, and be secondary to, the initial mitochondrial burst of superoxide formation [11, 14].

## Mitochondrial generation of reactive oxygen species

Reactive oxygen species generation by mitochondria has long been known to occur during both physiological and pathophysiological conditions. However, until recently, superoxide production during reperfusion was presumed to be the result of generalised dysregulation of the electron transport chain, with electrons leaking at multiple non-specific sites when oxygen was re-introduced to a system in biochemical disarray following a period of ischaemia [11].

**Fig. 1:**
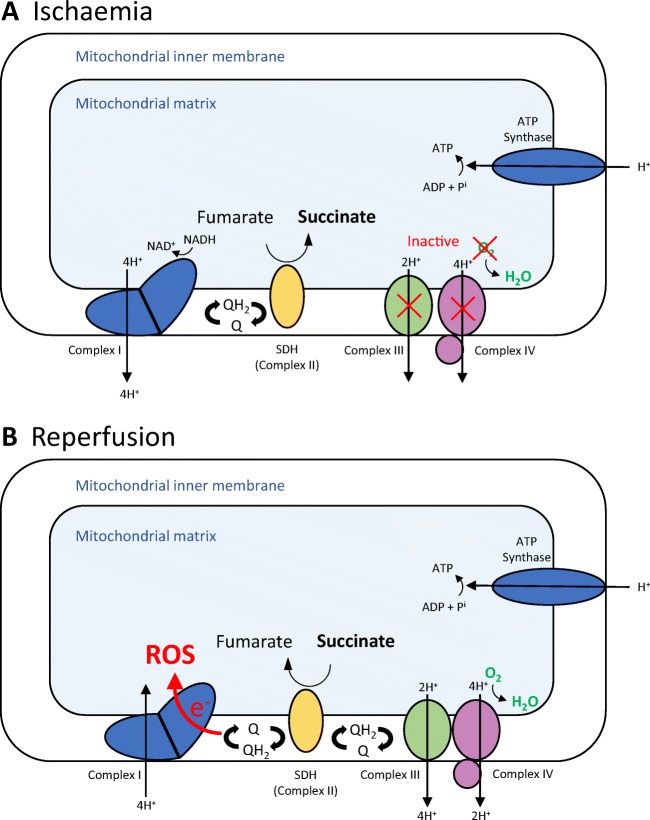
Mitochondrial generation of reactive oxygen species (ROS) during ischaemia reperfusion injury. Under normoxic conditions, the electron transport chain (ETC) transfers electrons from NADH and FADH_2_ to oxygen via a series of redox reactions. In this process, H^+^ is pumped out of the mitochondria generating a proton motive force. It is this proton motive that drives the production of energy, in the form of adenosine triphosphate (ATP), by ATP synthase. During ischaemia, without oxygen to accept electrons, the ETC rapidly ceases and the electron donors and carrier pools such as NADH and coenzyme Q (CoQ) become maximally reduced. Mitochondria briefly compensate for this by the oxidation of fumarate to succinate thereby replenishing the reduced carrier pools but generating a pool of succinate in the process. On reperfusion, the succinate that accumulates during ischaemia is rapidly oxidised maintaining a reduced CoQ pool and an environment that favours reverse electron transport (RET) and the generation of reactive oxygen species (superoxide)

Contrary to this view, Chouchani et al. recently identified a specific metabolic pathway in which superoxide was generated through reverse electron transport at complex I of the electron transport chain. Moreover, this process was shown to be driven by the pool of the citric acid cycle metabolite, succinate, that accumulates during ischaemia (Fig. **1**) [15].

This burst of mitochondrial superoxide leads to the activation of a plethora of pathways that cause tissue injury. Direct cellular or mitochondrial damage through lipid peroxidation or protein carbonylation results in disruption of adenosine triphospate (ATP) generation, dysregulation of calcium levels and induction of the mitochondrial permeability transition pore (MPTP) with subsequent activation of necrosis and apoptosis. Tissue damage can also occur indirectly through activation of the innate and adaptive immune response by damage-associated molecular patterns (DAMPs). These DAMPs can be cellular (e.g. high mobility group box 1 (HMGB1), hyaluronan [16]) or directly mitochondrial in origin. Mitochondrial ROS, generated by IR injury, have been shown to act directly as a DAMP. Furthermore, opening of the MPTP in response to mitochondrial dysfunction releases other mitochondrial DAMPs [17, 18] such as mtDNA, cytochrome *c*, succinate and N-formyl peptides [19, 20]. These processes provide a putative mechanism for the impact of mitochondrial dysfunction on longer-term renal function after an episode of AKI. The identification of a unifying, specific pathway also provides a potential explanation for the vast array of interventions and therapies that have previously been shown to ameliorate IR injury [11].

## Mitochondrial therapeutic strategies in IR injury

The recent progress in our understanding of the pathophysiological mitochondrial mechanisms that underpin IR injury has led to an array of potential applications of mitochondria as both targets for therapeutic strategies and as biomarkers of disease severity. The therapeutic strategies can be grouped into the following areas and are discussed in more detail below: limiting oxidative stress and mitochondrial ROS generation, reducing tubular cell death through necrosis and apoptosis, moderating mitochondrial dynamics and mitochondrial immunomodulation.

### Mitochondria oxidative stress

The fundamental role of mitochondrial oxidative stress in a wide range of pathologies including IR injury has been extensively reported in the literature and has provided a strong rationale for the use of antioxidants as a therapeutic intervention. Unfortunately, the clinical translation of non-specific antioxidants has been almost universally disappointing. This paradox can be interpreted in one of two ways. Either reactive oxygen species do not have a role in the pathophysiology of these diseases or the antioxidants are not adequately delivered to the appropriate region of the cell to prevent oxidative damage. This second argument is supported by the increasing recognition of the important physiological roles of ROS within the cell and the recognition of the integral role of the burst of mitochondrial ROS in mediating IR injury. Targeting antioxidants to mitochondria, therefore, provides an approach that could both explain this paradox and provide a novel therapeutic strategy in IR injury [5, 21].

Bioactive molecules and drugs, including antioxidants, have been targeted to mitochondria in vivo using both lipophilic cations and mitochondrial targeted peptides (reviewed in detail elsewhere [21]). Both these approaches lead to a rapid and significant accumulation of the targeted compound within the mitochondria. This approach increases potency whilst enabling a lower dose to be administered, minimising off-target effects and toxicity. Triphenylphosphonium (TPP) is a lipophilic cation that is rapidly taken up into mitochondria and concentrated several hundred-fold due to the large mitochondrial membrane potential in vivo*.* Covalent linkage of bioactive molecules or drugs to TPP has been used for a wide range of compounds [21–24]. The most extensively investigated of these is MitoQ. The bioactive molecule of MitoQ is ubiqinone. This is rapidly reduced in mitochondria to the chain-breaking antioxidant ubiquinol, which directly scavenges mitochondrial ROS, inhibiting downstream lipid peroxidation and mitochondrial damage. MitoQ has been shown to protect against oxidative injury in a variety of animal models and has been used in phase II human trials [25, 26]. In models of renal IR injury, MitoQ has recently been demonstrated to reduce markers of oxidative injury, renal function and tissue injury following IR injury [27–29].

Another approach is through the use of peptide delivery systems including the Szeto-Schiller (SS) peptides and the mitochondrial penetrating peptides. The precise mechanism of action of these molecules is not understood, but they have been proposed to protect mitochondria by interacting with cardiolipin [30]. SS peptides have also been shown to ameliorate renal IR injury in rodents and have been studied in other models of IR pathologies [31]. The lead compound in this group, SS-31, has been investigated in larger animal models and is currently the subject of a human clinical trial investigating its efficacy in ameliorating IR injury post-angioplasty for renal artery stenosis [32].

**Fig. 2:**
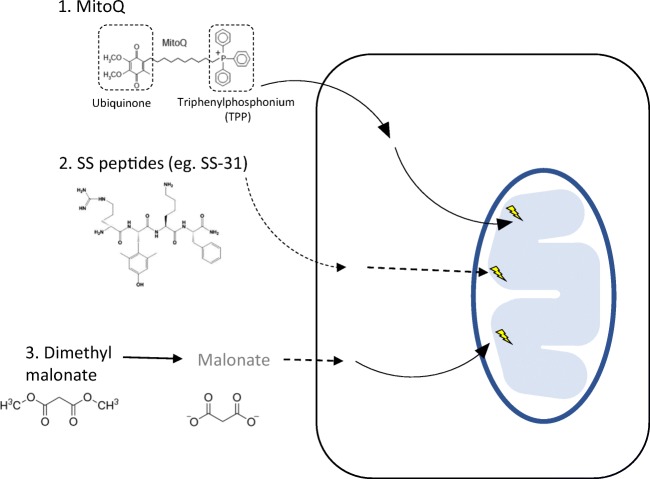
Mitochondrial agents targeting reactive oxygen species in ischaemia reperfusion. A number of approaches have been investigated in vivo to target mitochondrial reactive oxygen species (ROS) during ischaemia reperfusion (IR) injury. **1**. Triphenylphosponium (TPP) is rapidly taken up into mitochondria and concentrated several hundred-fold. Bioactive molecules can be covalently linked to TPP thus enabling the selective, rapid uptake of these molecules into mitochondria. MitoQ is an example of this approach. The bioactive molecule of MitoQ is ubiquinone. This is a chain breaking antioxidant that directly scavenges mtROS thereby preventing downstream tissue damage. **2**. The precise mechanism of Szeto-Schiller (SS) peptides is less well characterised but it is thought to interact with cardiolipin. They have demonstrated efficacy in a range of models in reducing IR injury. **3**. The small molecule competitive inhibitor of succinate dehydrogenase, malonate, has been shown to reduce IR injury in a range of in vivo models. Dimethyl malonate can be administered intravenously and is rapidly hydrolysed to malonate. Malonate rapidly diffuses across the cellular and mitochondrial membranes where it can then competitively inhibit succinate dehydrogenase and reduce the accumulation of succinate during ischaemia and IR injury

The recognition of a specific metabolic pathway that drives mitochondrial ROS production during IR injury opens up the possibility of a novel therapeutic strategy that acts upstream of ROS generation [15], namely competitive inhibition of succinate dehydrogenase, which has been shown to ameliorate IR injury in a variety of in vivo models [15, 33]. The metabolic signature of ischaemic succinate accumulation has been demonstrated in a wide range of tissues including human myocardial [34] and renal tissue [35]. Contrary to these findings, some authors have questioned the translation of these findings in small animals to human tissues [36]. An inter-species difference in mechanisms of mitochondrial ROS generation is unlikely, given the very early evolutionary origin of mitochondria, and it is essential to rule out differences in experimental methodology as a source of conflicting data. Therefore, preventing succinate accumulation by inhibiting succinate dehydrogenase activity remains a potentially important, but as yet unexplored, area of therapy in renal IR injury. Furthermore, the demonstrated therapeutic potential in other models of IR injury make it an appealing mechanism that warrants future investigation (Fig. **2**).

### Tubular death through apoptosis and necrosis

Mitochondria are recognised to be integral to the processes of necrosis and apoptosis which underlie tubular injury and cell death following IR injury [12]. In mammalian cells, apoptosis is initiated through two major but interconnected pathways: death receptors and mitochondria. The mitochondrial pathway is characterised by an increase in the permeability of the outer mitochondrial membrane (MOMP) with the release of pro-apoptotic factors such as cytochrome *c*. The B-cell lymphoma 2 (Bcl-2) family proteins are important regulators of MOMP, both in a positive and negative capacity [37]. The release of cytochrome *c*, and other proteins, from the intermembrane space triggers the formation of the apoptosome which consists of cytochrome *c*, apaf-1 and caspase-9. This then activates downstream caspase-activation pathways resulting in apoptosis. However, despite the potential promise of therapies designed to inhibit apoptosis in AKI, it has yet to be realised in routine clinical practice [38]. A promising approach that is currently in phase II human clinical trials to treat AKI is the use of a small interfering RNA (siRNA) that temporarily inhibits expression of the stress response gene p53 [39, 40]. Other approaches that target the same pathway include disrupting steps in the apoptotic pathway using small molecules, caspase inhibitors and recombinant proteins [12].

### Mitochondrial biogenesis, mitophagy and dynamics

Mitochondria are highly dynamic organelles existing not as solitary, isolated entities but as a complex, interconnected network that undergoes continuous biogenesis, fusion, fission and the selective removal by autophagy, termed mitophagy. These processes are all essential for normal mitochondrial and cellular function [41, 42] and have been shown to be implicated in AKI.

Altered mitochondrial dynamics contributes to changes in mitochondrial energetics, cellular injury and repair following AKI [42]. A variety of mammalian proteins have been identified as regulators of mitochondrial fission and fusion including the pro-fusion proteins, mitofusin 1 and 2, and OPA1, and the pro-fission protein dynamin-related protein1 (DRP1) [5, 43, 44]. The activation of DRP1 results in the translocation of DRP1 to the outer mitochondrial membrane promoting mitochondrial fission and exacerbating AKI. Pharmacological inhibition of DRP1 in a mouse model of AKI reduces mitochondrial fission and ameliorates AKI in vivo [45]. Sirtuin 3 (SIRT3) has also been shown to have a functional role in mitochondrial dynamics, preserving mitochondrial integrity by preventing DRP1 translocation. SIRT3 upregulation was shown to be protective in vitro in human proximal tubular epithelial cells damaged by cisplatin. Furthermore, in a murine model of AKI, upregulation of SIRT3 resulted in a decrease in mitochondrial fission whilst SIRT3 deficiency in the *Sirt3*^*−/−*^ mice exacerbated cisplatin-induced AKI [46].

In the murine kidney, mitophagy has been shown to be highly active [47, 48], with an integral role in moderating tissue injury in the kidney. Ablation of key genes that regulate autophagy, such as autophagy-related protein 7 (ATG7) and ATG5, has been shown to exacerbate AKI in vivo [48, 49]. There is also evidence that there is crosstalk between the cell death machinery and mitophagy. Deletion of the pro-apoptotic protein BAK has been shown to be reno-protective in models of IR injury. This reno-protective effect was associated with a decrease in the release of cytochrome *c* and mitochondrial fragmentation [37].

Following AKI, the resolution of kidney injury and return of function is primarily through restoration of cellular function rather than regeneration and cell proliferation. Therefore, moderating mitochondrial biogenesis may provide another therapeutic avenue in AKI [42, 50]. Peroxisome proliferator-activated receptor-γ coactivator-1α (PGC-1α) has been identified as a key regulator of mitochondrial biogenesis and PGC-1α knock-out mice have been shown to be more susceptible to kidney injury [51]. More recently, sirtuins have also been shown to have regulatory roles in mitochondria biogenesis, and the SIRT1 activator SRT1720 has been shown to augment mitochondrial recovery and tubular function in the rat in vivo following IR injury [52]. There is also emerging evidence that the β_2_-antagonist formoterol has effects as an activator of mitochondrial biogenesis and can enhance recovery of mitochondria and kidney function following IR injury [5, 53].

### Mitochondrial immunomodulation

Mitochondria are increasingly being recognised as having critical roles in activating and moderating the immune system though a range of pathways [17, 54, 55]. In adult kidney transplantation, there is evidence to suggest that prolonged cold ischaemia impacts on long-term graft survival. Despite significant improvements in short-term outcomes, the prevalence of chronic allograft dysfunction remains largely unchanged [56]. The emerging evidence that mitochondria are not only integral to IR injury but also have fundamental roles in the immune response suggests there may be new avenues to improve long-term graft outcomes. Mitochondria, for example, are thought to be involved in regulation of conversion of M1 inflammatory macrophages to M2 anti-inflammatory cells. Both metformin and rotenone have been shown to facilitate this switch, possibly mediated through actions on complex I by reducing reverse electron transport and ROS [17, 57, 58]. Furthermore, the inhibition of ROS generation by inhibiting SDH has also been shown to limit pro-inflammatory responses and boost anti-inflammatory responses [58, 59].

Recently, it has also been suggested that maladaptive repair following AKI may be responsible for the progression of renal disease and development of chronic kidney disease in affected individuals [60]. Interestingly, the cellular changes underlying maladaptive repair, most notably cellular senescence in tubular epithelial cells and adoption of a pro-fibrotic phenotype, mimic those of kidney ageing [60, 61].

## Mitochondrial DNA release as a biomarker of kidney injury

**Fig. 3:**
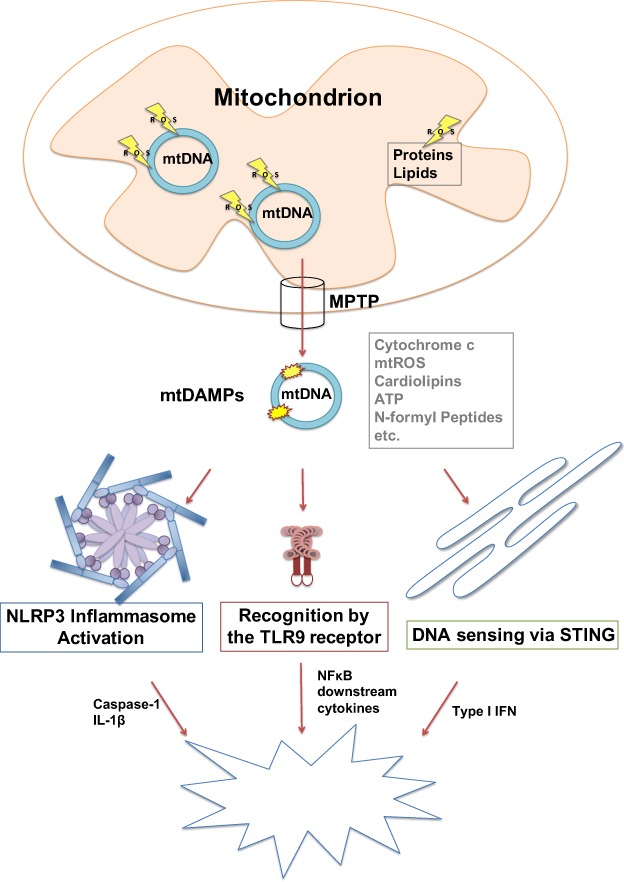
The role of mtDNA in activating the innate immune response. Ischaemic damage-induced loss of membrane potential and mitochondrial swelling can lead to opening of a non-selective permeability transition pore (MPTP) in the mitochondrial inner membrane that releases mitochondrial molecules, like cytochrome *c*, ATP, ROS, *N*-formyl peptides and mtDNA, into the cytosol. Released mitochondrial components can act as mitochondrial damage associated molecular patterns (mtDAMPs). When released, they function as signals for injury in cells and activate the innate immune response. MtDNA, due to its similarity to bacterial DNA, can activate the TLR9 dependent immune response and in turn stimulate activation of the transcription factor NF-κB and therefore expression of the cytokine IL-6, which is released from the cell and functions as a stimulus for immune cells. In addition, mtDNA is involved in the activation of the NLRP3 inflammasome that senses cytosolic DNA and stimulates the caspase-1-dependent release of IL-1β and IL-18. Furthermore, the ER-linked STING pathway can be activated inducing the expression of interferon type 1 and its inflammatory signalling pathways

The damage to mitochondria associated with IR injury results in the opening of the MPTP with the consequent release of a number of mitochondrial components into the cytosol. These function as DAMPs, activating an innate immune response and driving the systemic inflammatory response associated with IR injury. mtDNA is an example of such a DAMP (Fig. 3). A number of studies have examined the role of circulating mtDNA in the blood of patients. The levels of mtDNA have been shown to increase in the circulation following trauma [18] and more recently have been shown to correlate with mortality in intensive care unit patients [62]. It has also been investigated in the clinical context of severe lung injury and AKI, and shows promise as a potential plasma biomarker [27, 62–67]. Increased plasma mtDNA levels can be found in murine models of AKI and have been shown to be linked to an elevated innate immune response via activation of the TLR9 pathway [68]. In addition to its detection in the circulation, mtDNA can also be detected in the urine and levels have been shown to be significantly increased in the urine of patients after AKI [69–72]. The recognition that the kidney has a role in the clearance of DAMPs from plasma and the efficacy of dialysis in decreasing the amount of circulating mtDNA in patients may have future implications for our understanding of the pathogenesis of IR injury in AKI and the role of dialysis in managing IR injury [73–75].

## Summary

AKI remains a major problem in children and IR injury is either the primary aetiology or is implicated in a significant proportion of cases. Mitochondrial dysfunction or damage are increasingly recognised as fundamental to IR injury, generating the burst of ROS that initiates downstream tissue injury. They also have key roles in a variety of downstream processes, including the direct activation of the innate immune response, immunomodulation, and apoptosis and necrosis. Plasma and urinary mtDNA may have roles as biomarkers of IR injury in the future and there are a number of therapeutic strategies that are being explored to ameliorate mitochondrial dysfunction. We look forward with interest to the translation of these promising strategies to the management of AKI in children in the future.

## References

[1] Belayev LY, Palevsky PM (2014). The link between acute kidney injury and chronic kidney disease. Curr Opin Nephrol Hypertens.

[2] Coca SG, Singanamala S, Parikh CR (2012). Chronic kidney disease after acute kidney injury: a systematic review and meta-analysis. Kidney Int.

[3] Kaddourah A, Basu RK, Bagshaw SM, Goldstein SL, Investigators AWARE (2017). Epidemiology of acute kidney injury in critically ill children and young adults. N Engl J Med.

[4] Ullmann LP (1981). Cognitions: help or hindrance?. J Behav Ther Exp Psychiatry.

[5] Hall AM, Schuh CD (2016). Mitochondria as therapeutic targets in acute kidney injury. Curr Opin Nephrol Hypertens.

[6] Eltzschig HK, Eckle T (2011). Ischemia and reperfusion—from mechanism to translation. Nat Med.

[7] Jennings RB, Sommers HM, Smyth GA, Flack HA, Linn H (1960). Myocardial necrosis induced by temporary occlusion of a coronary artery in the dog. Arch Pathol.

[8] Braunwald E, Kloner RA (1985). Myocardial reperfusion: a double-edged sword?. J Clin Invest.

[9] Hearse DJ, Humphrey SM, Bullock GR (1978). The oxygen paradox and the calcium paradox: two facets of the same problem?. J Mol Cellm Cardiol.

[10] Raedschelders K, Ansley DM, Chen DD (2012). The cellular and molecular origin of reactive oxygen species generation during myocardial ischemia and reperfusion. Pharmacol Ther.

[11] Chouchani ET, Pell VR, James AM, Work LM, Saeb-Parsy K, Frezza C, Krieg T, Murphy MP (2016). A unifying mechanism for mitochondrial superoxide production during ischemia-reperfusion injury. Cell Metab.

[12] Padanilam BJ (2003). Cell death induced by acute renal injury: a perspective on the contributions of apoptosis and necrosis. Am J Physiol Renal Physiol.

[13] Kezic A, Stajic N, Thaiss F (2017). Innate immune response in kidney ischemia/reperfusion injury: potential target for therapy. J Immunol Res.

[14] Jassem W, Fuggle SV, Rela M, Koo DD, Heaton ND (2002). The role of mitochondria in ischemia/reperfusion injury. Transplantation.

[15] Chouchani ET, Pell VR, Gaude E, Aksentijevic D, Sundier SY, Robb EL, Logan A, Nadtochiy SM, Ord EN, Smith AC, Eyassu F, Shirley R, Hu CH, Dare AJ, James AM, Rogatti S, Hartley RC, Eaton S, Costa ASH, Brookes PS, Davidson SM, Duchen MR, Saeb-Parsy K, Shattock MJ, Robinson AJ, Work LM, Frezza C, Krieg T, Murphy MP (2014). Ischaemic accumulation of succinate controls reperfusion injury through mitochondrial ROS. Nature.

[16] Braza F, Brouard S, Chadban S, Goldstein DR (2016). Role of TLRs and DAMPs in allograft inflammation and transplant outcomes. Nat Rev Nephrol.

[17] Mills EL, Kelly B, O'Neill LAJ (2017). Mitochondria are the powerhouses of immunity. Nat Immunol.

[18] Zhang Q, Raoof M, Chen Y, Sumi Y, Sursal T, Junger W, Brohi K, Itagaki K, Hauser CJ (2010). Circulating mitochondrial DAMPs cause inflammatory responses to injury. Nature.

[19] Krysko DV, Agostinis P, Krysko O, Garg AD, Bachert C, Lambrecht BN, Vandenabeele P (2011). Emerging role of damage-associated molecular patterns derived from mitochondria in inflammation. Trends Immunol.

[20] Kang JW, Kim SJ, Cho HI, Lee SM (2015). DAMPs activating innate immune responses in sepsis. Ageing Res Rev.

[21] Smith RA, Hartley RC, Cocheme HM, Murphy MP (2012). Mitochondrial pharmacology. Trends Pharmacol Sci.

[22] Chouchani ET, Methner C, Nadtochiy SM, Logan A, Pell VR, Ding S, James AM, Cocheme HM, Reinhold J, Lilley KS, Partridge L, Fearnley IM, Robinson AJ, Hartley RC, Smith RA, Krieg T, Brookes PS, Murphy MP (2013). Cardioprotection by S-nitrosation of a cysteine switch on mitochondrial complex I. Nat Med.

[23] Arndt S, Baeza-Garza CD, Logan A, Rosa T, Wedmann R, Prime TA, Martin JL, Saeb-Parsy K, Krieg T, Filipovic MR, Hartley RC, Murphy MP (2017). Assessment of H2S in vivo using the newly developed mitochondria-targeted mass spectrometry probe MitoA. J Biol Chem.

[24] Logan A, Pell VR, Shaffer KJ, Evans C, Stanley NJ, Robb EL, Prime TA, Chouchani ET, Cochemé HM, Fearnley IM, Vidoni S, James AM, Porteous CM, Partridge L, Krieg T, Smith RA, Murphy MP (2016). Assessing the mitochondrial membrane potential in cells and in vivo using targeted click chemistry and mass spectrometry. Cell Metab.

[25] Gane EJ, Weilert F, Orr DW, Keogh GF, Gibson M, Lockhart MM, Frampton CM, Taylor KM, Smith RA, Murphy MP (2010). The mitochondria-targeted anti-oxidant mitoquinone decreases liver damage in a phase II study of hepatitis C patients. Liver Int.

[26] Smith RA, Murphy MP (2010). Animal and human studies with the mitochondria-targeted antioxidant MitoQ. Ann N Y Acad Sci.

[27] Dare AJ, Logan A, Prime TA, Rogatti S, Goddard M, Bolton EM, Bradley JA, Pettigrew GJ, Murphy MP, Saeb-Parsy K (2015). The mitochondria-targeted anti-oxidant MitoQ decreases ischemia-reperfusion injury in a murine syngeneic heart transplant model. J Heart Lung Transplant.

[28] Liu X, Murphy MP, Xing W, Wu H, Zhang R, Sun H (2018). Mitochondria-targeted antioxidant MitoQ reduced renal damage caused by ischemia-reperfusion injury in rodent kidneys: Longitudinal observations of T2-weighted imaging and dynamic contrast-enhanced MRI. Magn Reson Med.

[29] Dare AJ, Bolton EA, Pettigrew GJ, Bradley JA, Saeb-Parsy K, Murphy MP (2015). Protection against renal ischemia-reperfusion injury in vivo by the mitochondria targeted antioxidant MitoQ. Redox Biol.

[30] Birk AV, Liu S, Soong Y, Mills W, Singh P, Warren JD, Seshan SV, Pardee JD, Szeto HH (2013). The mitochondrial-targeted compound SS-31 re-energizes ischemic mitochondria by interacting with cardiolipin. J Am Soc Nephrol.

[31] Szeto HH, Liu S, Soong Y, Alam N, Prusky GT, Seshan SV (2016). Protection of mitochondria prevents high-fat diet-induced glomerulopathy and proximal tubular injury. Kidney Int.

[32] Saad A, Herrmann SMS, Eirin A, Ferguson CM, Glockner JF, Bjarnason H, McKusick MA, Misra S, Lerman LO, Textor SC (2017) Phase 2a clinical trial of mitochondrial protection (Elamipretide) during stent revascularization in patients with atherosclerotic renal artery stenosis. Circ Cardiovasc Interv 10(9). doi: 10.1161/CIRCINTERVENTIONS.117.005487 10.1161/CIRCINTERVENTIONS.117.005487 PMC5659347 28916603

[33] Valls-Lacalle L, Barba I, Miro-Casas E, Alburquerque-Bejar JJ, Ruiz-Meana M, Fuertes-Agudo M, Rodriguez-Sinovas A, Garcia-Dorado D (2016). Succinate dehydrogenase inhibition with malonate during reperfusion reduces infarct size by preventing mitochondrial permeability transition. Cardiovasc Res.

[34] Kohlhauer M, Dawkins S, Costa ASH, Lee R, Young T, Pell VR, Choudhury RP, Banning AP, Kharbanda RK; Oxford Acute Myocardial Infarction (OxAMI) Study, Saeb-Parsy K, Murphy MP, Frezza C, Krieg T, Channon KM (2018) Metabolomic profiling in acute ST-segment-elevation myocardial infarction identifies succinate as an early marker of human ischemia-reperfusion injury. J Am Heart Assoc 7(8). DOI: 10.1161/JAHA.117.007546 10.1161/JAHA.117.007546 PMC6015393 29626151

[35] (2017) Abstracts of the 18th Congress of the European Society for Organ Transplantation, 24–27 September 2017, Barcelona, Spain. Transplant Int 30(Suppl 2):5–576 10.1111/tri.13072 28944506

[36] Wijermars LG, Schaapherder AF, Kostidis S, Wust RC, Lindeman JH (2016). Succinate accumulation and ischemia-reperfusion injury: of mice but not men, a study in renal ischemia-reperfusion. Am J Transplant.

[37] Wei Q, Dong G, Chen JK, Ramesh G, Dong Z (2013). Bax and Bak have critical roles in ischemic acute kidney injury in global and proximal tubule-specific knockout mouse models. Kidney Int.

[38] Nicholson DW (2000). From bench to clinic with apoptosis-based therapeutic agents. Nature.

[39] Titze-de-Almeida R, David C, Titze-de-Almeida SS (2017). The race of 10 synthetic RNAi-based drugs to the pharmaceutical market. Pharm Res.

[40] Demirjian S, Ailawadi G, Polinsky M, Bitran D, Silberman S, Shernan SK, Burnier M, Hamilton M, Squiers E, Erlich S, Rothenstein D, Khan S, Chawla LS (2017). Safety and tolerability study of an intravenously administered small interfering ribonucleic acid (siRNA) post on-pump cardiothoracic surgery in patients at risk of acute kidney injury. Kidney Int Rep.

[41] Song M, Franco A, Fleischer JA, Zhang L, Dorn GW (2017). Abrogating mitochondrial dynamics in mouse hearts accelerates mitochondrial senescence. Cell Metab.

[42] Bhargava P, Schnellmann RG (2017). Mitochondrial energetics in the kidney. Nat Rev Nephrol.

[43] Chen H, Chan DC (2009). Mitochondrial dynamics—fusion, fission, movement, and mitophagy—in neurodegenerative diseases. Hum Mol Genet.

[44] Zhan M, Brooks C, Liu F, Sun L, Dong Z (2013). Mitochondrial dynamics: regulatory mechanisms and emerging role in renal pathophysiology. Kidney Int.

[45] Brooks C, Wei Q, Cho SG, Dong Z (2009). Regulation of mitochondrial dynamics in acute kidney injury in cell culture and rodent models. J Clin Invest.

[46] Morigi M, Perico L, Rota C, Longaretti L, Conti S, Rottoli D, Novelli R, Remuzzi G, Benigni A (2015). Sirtuin 3-dependent mitochondrial dynamic improvements protect against acute kidney injury. J Clin Invest.

[47] McWilliams TG, Prescott AR, Allen GF, Tamjar J, Munson MJ, Thomson C, Muqit MM, Ganley IG (2016). Mito-QC illuminates mitophagy and mitochondrial architecture in vivo. J Cell Biol.

[48] Kimura T, Isaka Y, Yoshimori T (2017). Autophagy and kidney inflammation. Autophagy.

[49] Jiang M, Wei Q, Dong G, Komatsu M, Su Y, Dong Z (2012). Autophagy in proximal tubules protects against acute kidney injury. Kidney Int.

[50] Rosen S, Stillman IE (2008). Acute tubular necrosis is a syndrome of physiologic and pathologic dissociation. J Am Soc Nephrol.

[51] Tran M, Tam D, Bardia A, Bhasin M, Rowe GC, Kher A, Zsengeller ZK, Akhavan-Sharif MR, Khankin EV, Saintgeniez M, David S, Burstein D, Karumanchi SA, Stillman IE, Arany Z, Parikh SM (2011). PGC-1alpha promotes recovery after acute kidney injury during systemic inflammation in mice. J Clin Invest.

[52] Funk JA, Schnellmann RG (2013). Accelerated recovery of renal mitochondrial and tubule homeostasis with SIRT1/PGC-1alpha activation following ischemia-reperfusion injury. Toxicol Appl Pharmacol.

[53] Jesinkey SR, Funk JA, Stallons LJ, Wills LP, Megyesi JK, Beeson CC, Schnellmann RG (2014). Formoterol restores mitochondrial and renal function after ischemia-reperfusion injury. J Am Soc Nephrol.

[54] West AP, Shadel GS, Ghosh S (2011). Mitochondria in innate immune responses. Nat Rev Immunol.

[55] Simmons JD, Lee YL, Mulekar S, Kuck JL, Brevard SB, Gonzalez RP, Gillespie MN, Richards WO (2013). Elevated levels of plasma mitochondrial DNA DAMPs are linked to clinical outcome in severely injured human subjects. Ann Surg.

[56] Libby P, Pober JS (2001). Chronic rejection. Immunity.

[57] Kelly B, Tannahill GM, Murphy MP, O'Neill LA (2015). Metformin inhibits the production of reactive oxygen species from NADH:ubiquinone oxidoreductase to limit induction of interleukin-1beta (IL-1beta) and boosts Interleukin-10 (IL-10) in lipopolysaccharide (LPS)-activated macrophages. J Biol Chem.

[58] Mills EL, Kelly B, Logan A, Costa ASH, Varma M, Bryant CE, Tourlomousis P, Däbritz JHM6, Gottlieb E6, Latorre I, Corr SC, McManus G, Ryan D, Jacobs HT, Szibor M, Xavier RJ, Braun T, Frezza C, Murphy MP, O'Neill LA (2016). Succinate dehydrogenase supports metabolic repurposing of mitochondria to drive inflammatory macrophages. Cell.

[59] Mills EL, Ryan DG, Prag HA, Dikovskaya D, Menon D, Zaslona Z, Jedrychowski MP, Costa ASH, Higgins M, Hams E, Szpyt J, Runtsch MC, King MS, McGouran JF, Fischer R, Kessler BM, McGettrick AF, Hughes MM, Carroll RG, Booty LM, Knatko EV, Meakin PJ, Ashford MLJ, Modis LK, Brunori G, Sévin DC, Fallon PG, Caldwell ST, Kunji ERS, Chouchani ET, Frezza C, Dinkova-Kostova AT, Hartley RC, Murphy MP, O'Neill LA (2018). Itaconate is an anti-inflammatory metabolite that activates Nrf2 via alkylation of Keap1. Nature.

[60] Ferenbach DA, Bonventre JV (2015). Mechanisms of maladaptive repair after AKI leading to accelerated kidney ageing and CKD. Nat Rev Nephrol.

[61] Yang L, Besschetnova TY, Brooks CR, Shah JV, Bonventre JV (2010). Epithelial cell cycle arrest in G2/M mediates kidney fibrosis after injury. Nat Med.

[62] Nakahira K, Kyung SY, Rogers AJ, Gazourian L, Youn S, Massaro AF, Quintana C, Osorio JC, Wang Z, Zhao Y, Lawler LA, Christie JD, Meyer NJ, Mc Causland FR, Waikar SS, Waxman AB, Chung RT, Bueno R, Rosas IO, Fredenburgh LE, Baron RM, Christiani DC, Hunninghake GM, Choi AM (2013). Circulating mitochondrial DNA in patients in the ICU as a marker of mortality: derivation and validation. PLoS Med.

[63] Gan L, Chen X, Sun T, Li Q, Zhang R, Zhang J, Zhong J (2015). Significance of serum mtDNA concentration in lung injury induced by hip fracture. Shock.

[64] Ellinger J, Muller DC, Muller SC, Hauser S, Heukamp LC, von Ruecker A, Bastian PJ, Walgenbach-Brunagel G (2012). Circulating mitochondrial DNA in serum: a universal diagnostic biomarker for patients with urological malignancies. Urol Oncol.

[65] Yu M (2012). Circulating cell-free mitochondrial DNA as a novel cancer biomarker: opportunities and challenges. Mitochondrial DNA.

[66] Czajka A, Ajaz S, Gnudi L, Parsade CK, Jones P, Reid F, Malik AN (2015). Altered mitochondrial function, mitochondrial DNA and reduced metabolic flexibility in patients with diabetic nephropathy. EBioMedicine.

[67] Lee HK, Song JH, Shin CS, Park DJ, Park KS, Lee KU, Koh CS (1998). Decreased mitochondrial DNA content in peripheral blood precedes the development of non-insulin-dependent diabetes mellitus. Diabetes Res Clin Pract.

[68] Tsuji N, Tsuji T, Ohashi N, Kato A, Fujigaki Y, Yasuda H (2016). Role of mitochondrial DNA in septic AKI via toll-like receptor 9. J Am Soc Nephrol.

[69] Pisareva NL (1982). Neurophysiologic analysis of the development of cortico-striatal connections during postnatal ontogeny in the rabbit. Neirofiziologiia.

[70] Eirin A, Saad A, Tang H, Herrmann SM, Woollard JR, Lerman A, Textor SC, Lerman LO (2016). Urinary mitochondrial DNA copy number identifies chronic renal injury in hypertensive patients. Hypertension.

[71] Jansen MPB, Pulskens WP, Butter LM, Florquin S, Juffermans NP, Roelofs J, Leemans JC (2018). Mitochondrial DNA is released in urine of SIRS patients with acute kidney injury and correlates with severity of renal dysfunction. Shock.

[72] Ho PW, Pang WF, Luk CC, Ng JK, Chow KM, Kwan BC, Li PK, Szeto CC (2017). Urinary mitochondrial DNA level as a biomarker of acute kidney injury severity. Kidney Dis (Basel).

[73] Wang YC, Lee WC, Liao SC, Lee LC, Su YJ, Lee CT, Chen JB (2011). Mitochondrial DNA copy number correlates with oxidative stress and predicts mortality in nondiabetic hemodialysis patients. J Nephrol.

[74] Cao H, Ye H, Sun Z, Shen X, Song Z, Wu X, He W, Dai C, Yang J (2014). Circulatory mitochondrial DNA is a pro-inflammatory agent in maintenance hemodialysis patients. PLoS One.

[75] Rao M, Li L, Demello C, Guo D, Jaber BL, Pereira BJ, Balakrishnan VS, HEMO Study Group (2009). Mitochondrial DNA injury and mortality in hemodialysis patients. J Am Soc Nephrol.

